# Valedictory Address to the Graduating Class of the Ohio College of Dental Surgery—Session of 1849–50

**Published:** 1850-04

**Authors:** George Mendenhall

**Affiliations:** Professor of Pathology and Therapeutics


DENTAL REGISTER OF THE WEST.

VOL. III. APRIL, 1850. NO. 3.

### VALEDICTORY ADDRESS TO THE GRADUATING CLASS OF THE OHIO COLLEGE OF DENTAL SURGERY—SESSION OF 1849-50.

BY GEORGE MENDENHALL, M. D.,

Professor of Pathology and Therapeutics.

Gentlemen: It has been a custom in this Institution, since its establishment, that one of the Faculty should at the annual commencement deliver a brief address to the graduating class. The object of this custom seems to be two-fold. In the first place, it gives an opportunity on behalf of the Faculty for a renewed expression of friendly feeling and interest in your welfare, and to assure you that this feeling is rendered more lively at the present time, because, by this last official act, the relation of teacher and pupil, which has heretofore been a strong tie between us, is to be severed. There is no time, perhaps, so well calculated to call forth expressions of friendly regard as occasions of this kind.

In the second place, there is no time so well suited to the purpose of indicating to you a few hints respecting your future professional course. The assiduous manner in which you have pursued your studies is an earnest to us that a few words of parting advice will be kindly received.

A period in your professional career has now been attained, which you once perhaps looked upon as almost the ultimatum of your hopes and expectations. You entered the field with an energy proportioned to the labor before you. Your industry has carried you along, first over one difficulty and then another, until the progress attained entitles you to a testimonial of merit and acquirement. These difficulties, no doubt, sometimes appeared to rise in your path mountain high, and threaten a complete obstruction to your future progress. They have so far been overcome, and you now pass into the world fully grown into professional manhood—not matured, it is true, but with the energy and activity of youth, and now requiring only a steady, straight-forward pursual in the line of professional duty, to develop fruit which you can bring to the altar of science, and add something valuable to that which is already known and appreciated.

The position you have now assumed is a new one—the relations which have surrounded you heretofore are now changed; you are no longer knocking at the portals of the profession of your choice, but you are admitted into it, and new duties and responsibilities claim your attention: to perform and discharge these well, is a matter of vast moment, not only to yourselves, but to your fellow beings. This profession is one involving the life and happiness of persons who, like yourselves, are seeking to avoid and be rid of the miseries incident to the imperfect condition of our organization. Man, in his primitive, rude, and simple state, required but little aid from medicine. Originally created in a state of mental and physical perfection, derangements in his functions and organization could not occur. But disobedience to the laws of God brought with it a train of liabilities to a thousand ills, some of which were inflicted at once on the authors of our sin and imperfection, others were left for gradual development, in accordance with the laws of fallen human organization, when acted upon by the causes of disease. The causes tending to *increase* this depravation are all around us; some of them cannot be *resisted*; but a portion at least of their influence may be *averted*, by placing the system under favorable circumstances of resistance to morbific agents, by keeping all of the functions of the body as nearly as possible in an equable state, and by timely repairing every breach made upon the citadel of life, however small this may be. We are are all aware that even a slight derangement of structure or function predisposes to other diseases, which cannot fail to appear when causes are applied calculated to excite them. We know something, also, respecting the effects of the imperfect performance of a function in affecting dependent functions, and thus the predispositions and actual causes of diseases become indefinitely multiplied. To avoid the effects of those causes of disease from which we cannot escape, is both our interest and our duty. We are surrounded too, by a thousand influences that *may* be avoided, but, when not avoided, assail this frail tenement of man's habitation at all points. Experience teaches us that we are all constantly trangressing the laws of our organization; that we are not only not avoiding influences to which I have alluded, but that we are every day voluntarily subjecting ourselves to them. Thus we are adding transgression of our own, with its effects, to the transgression of our progenitors, and in this way accumulating physical and moral evils upon ourselves, by adding to the degeneracy of our organization, and transmitting it to posterity in a still more degraded condition than we received it from our parents. Surely, transgression of the laws of our Maker is not confined to our first parents; neither are the effects with us confined to the guilty transgressor. How immeasurably then is our guilt increased by pursuing a course, the effects of which must be visited upon innocent victims!

Many of these evils have accumulated upon us while living in a state of what is called civilization. They have grown out of this condition, not necessarily, but by the abuses of the many privileges it affords. While incidentally, civilization has brought upon us many diseases, it is from this state of society that we have developed, and expect to develop still more, the means of preventing and curing them. The object of the profession of medicine is to avert and remedy these evils. Is not this a glorious object? and who among you does not feel himself nerved and stimulated to press forward until an exalted degree of excellence and usefulness is obtained, in accomplishing the purposes sought after? And let me here remind you, that you have been admitted this day into a department of this profession. The glory and shame of it is now in part yours. It is an honorable profession, and to the cultivation of which I trust you have this day pledged yourselves without mental reservation, to devote the remainder of your lives. In entering this profession and pledging yourselves to its advancement, I have remarked that you have new duties and responsibilities pressing upon you, which it is important should be rightly discharged. And what are these new obligations and responsibilties that you have assumed? And what privileges, also, does this new position give you?

In considering them, they may with propriety be divided into, 1st. Those obligations and privileges which pertain to the profession to which you now belong; 2d. The obligations and privileges that relate to the community in which you live.

The relations to the profession may be again divided into those which you sustain towards the general profession of medicine, and into those relating to the speciality to which you are more particularly attached.

I have said you were now a part of the medical profession; you belong to that class of persons who cultivate and practice the healing art; and as such, I am permitted to call your attention to a few leading ideas. If the glory and shame of this profession in part belong to you, you have an interest, an absorbing interest, in its condition, and are called upon to contribute your quota to its advancement, and to its preservation from those who would destructively lay their unholy hands upon this fabric. We would by no means claim for it perfection, but we do claim for it a respect and reverence, founded upon its intrinsic merits. It has had for its builders many minds of the highest order of intellectual qualifications, whose researches have stood the test of time—truths that are immutable enter into its structure. Its present condition is the result of the gradual growth of many centuries—fact after fact has been added and arranged, until now we are called upon to admire its beauty and usefulness. It is also mounted upon the car of progress, and is not a whit behind its sister sciences in the rapidity of its advancement towards that degree of perfection, which, in the nature of things, it is capable of assuming. Thousands are zealously laboring to clear away falsehood and error, so that the gems of truth may not be obscured. They are also developing truths new to us, and confirming those believed before—in short, a progressive spirit is abroad in the profession, and all its members should be thoroughly imbued with it. It has been charged upon the medical profession, that it is standing still—that there is no advancement; and there is an aversion and a spirit of intolerance to new things, and against those who labor for reform and advancement. Persons making this charge know not of what they speak, or they have a selfish motive in making the assertion. They must be utterly ignorant of our medical literature, or else void of honesty. Examine the pages of the numerous periodicals of this country and Europe, yes, and even of Asia, and you are at once struck with the advancing strides of our science and art. Then turn to the pages of our systematic works, and you there find the wheat separated from the chaff—the lighter portions have been removed, and truths, that are truths, are recorded in a more permanent form. Thus we proceed; active minds are constantly suggesting, theorizing, practicing, experimenting, and proving; the results are scattered abroad in our land; others subject the labors of these men to another ordeal; their opinions and practice are thus purified, until the few grains of pure gold found at the bottom are evolved, and carefully cherished by the profession. This examination will also show you that any and all kinds of opinion are tolerated, subject only to tests which relate to their truth or falsity. One condition only is necessary to entitle them to an examination, and this is, that no exclusive, dictatorial, boasting, or selfish spirit be exhibited—no high-sounding claims to superior knowledge, coupled with interested or sordid motives, can be tolerated in a liberal and high-minded profession like ours. These alleged improvements must be proposed in a spirit of becoming modesty, fully impressed with the idea that they *may* be wrong—that time and the experience of others, who can view them more dispassionately, may not verify their conclusions.

The nature of facts in medicine requires this, and no man loving his profession desires the triumph of opinions, unless they can be proven by *more* than one disinterested witness. After an examination of our literature which is teeming from the press, no candid man can say that the profession is standing still. The history of our profession shows a continued active state of progress, from the first dawn of medical truth until the present moment, and every indication points to a still more rapid progress, both as a science and an art. It has too much become the custom of the day to admit incompetent witnesses on the stand, to testify against the medical profession. They assert that there is no advancement in what is termed the regular profession—nothing new in it—everything belongs to a past age; while they, with an assurance equalled only by their want of knowledge, proclaim upon the house-top, theories and practices in disease as *new*, that have long been familiar to the profession, and which have, in many instances, been discarded as utterly worthless, or of minor importance. Some of them perhaps may be new, but whether true or false, evidences of their truth have entirely failed to be presented that can satisfy those used to the investigation of medical truths. These *pretended* reformers are legion, differing widely in their views, but all stoutly proclaiming the impossibility of error in their systems. Perfection in medicine has been attained; the philosopher's stone has been discovered; and death, the grim monster, will cease now to stalk abroad in the land, because *we* have discovered a universal panacea for every ill. This is a fair specimen of their boasting. These are the most prominent features of all the exclusive excrescences which cling to the profession, and the great object of which is the accumulation of wealth. Error may often be excused when connected with honesty of purpose; but learn always to suspect every man of great self-pretensions; and doubly so, if there is any selfish motive connected with it. Most of this high-sounding, pretended advancement is indeed a retrograde progression—a progression in ignorance.

But you may possibly hear it asserted that they tend to improve the profession, and that much which is valuable is drawn from these newly broached doctrines and practices. I admit the profession may be benefited by them, and so may we all draw instruction, if we choose, from the ignorance, follies, and depravity of those around us.

Now, while I would commend to your favorable consideration the cultivation of a spirit of improvement—to press on towards perfection, never resting satisfied with what you have attained—I would also caution you not to be too hasty in adopting new views; try, prove, and hold fast to that which is good. Never adopt that which is new, because it is new: neither reject it for the same reason alone.

We find, in examining the history of medicine, that a book was once written, dedicated to all those "who thought new things better than old, simply because they were new:" surely this would be an extensive dedication if done at this day. The history of the author of this book *should* be a warning to some modern pretenders not to take newness *alone* as an *evidence* of truth. The pathway of truth in science is a republican way, which all may tread, and the world always, sooner or later, awards to every man who travels it his just merits. If any are able to select something new and valuable, the meed of praise is seldom withheld; petty jealousies soon pass by and are forgotten; but when the *pretender* claims to press upon your notice his assumed discoveries, examine carefully whether they be true; watch the motive, and judge accordingly.

Under these circumstances, you are expected to promote the advancement, guard the honor of the profession, and to discountenance quackery in whatever form it may present itself. Perhaps it may occur to you, that because you belong to a speciality of the profession, you will allow the other portion to take care of itself. I warn you not to commit this error, or you may make the discovery of it only after you have perpetrated a serious error on the profession, including yourselves.

In the application of the term quackery, I do not mean here to designate any particular man or set of men by the term; and that I may not be misunderstood, I will define what I mean by the term. The verb to quack means, "to make a noisy crying or talking; to make noisy claims or pretensions." Quackery, then, means any system or thing which permits or practices quacking, noisy talking, or makes noisy pretensions. When this definition is applicable to one professing medical knowledge, then you have a medical quack. After defining what is meant by the term, I leave you to make the application for yourselves. If you find any man, or any body or association of men, that practice in the manner of my definition, then they are quacks. No matter whether they assume to practice the general profession of medicine or a speciality of that profession, but wherever and whenever found, shun them professionally as you would the breath of pestilence. From your position, it will be in your power to exert a great influence upon the profession, and correct many false impressions respecting it. Your evidence may be deemed of more value because you are not practicing the general branches of the profession; and it is your duty to give it in favor of the truth.

On the other hand, what privileges and courtesies have you a right to expect from the profession, now you are a faithful member of it. In the first place, you are entitled to an equality in every respect of companionship with medical men, and your professional opinions are entitled to equal con-consideration on all points of professional intercourse which are common to you both. In the general practice, the authority of their opinions will be superior to yours; while in the practice of the speciality of dentistry, your opinions will be entitled to the supremacy. These concessions will be mutual, neither claiming superiority over the other, and yet each superior in your own proper department, with an entire equality on ground which is common to you both. Another consideration you have a right to expect from the general profession is, that they will discountenance quackery in your speciality—that they will on all occasions give their influence to those capable and well educated dentists who are true to their profession. This is due to you as much as it is your duty to sustain the profession. These I conceive to be the proper relations you hold to the profession of medicine, and it to you as a portion of that profession.

In regard to the relations you hold towards those honorably practicing your speciality, the same general conditions must govern. While a fair and honorable competition is allowable, nothing can be tolerated which savors of the spirit of quackery. Every man, before he can expect your fraternal confidence, must practice his profession in an open, candid manner. He must make no exclusive, high-sounding, and noisy pretensions; possess no knowledge but what he is willing to communicate freely to others of the profession of medicine; and deal in no specifics or secret remedies, or plans of practice. This course you have a right to expect of others, and they have a right to demand the same of you. A contrary one must result in a want of confidence, and is mean, undignified, unmanly, dishonorable, and disgraceful in the highest degree. The indignation of every member of the profession should be visited on any man who arrogates to himself knowledge and modes of treating disease that he is not willing to scatter abroad among his professional brethren. A sordid spirit of that kind should be rebuked, no matter what qualifications its possessor may have. Every man, also, upon entering a profession, places himself under obligation to that profession to contribute to its common stock of knowledge, and to aid in its elevation and advancement by adding something to it not before known. Our whole science being progressive, we must assist in promoting its onward march—each in his proper department must exhibit a commendable spirit of industry. We must neither be too indolent, too cowardly, or too proud to aid in fulfilling the mission which destiny has marked out for that profession which has science for its foundation, and the good of mankind for its great object.

In the next place, what are the obligations and privileges that relate to the community in which you live? Or, in other words, what has the public a right to expect from you, and in return, what have you a right to expect from it?

You have before the world assumed the duties and responsibilities of a new profession—one in which each one of you say to the public, "I am prepared and qualified to serve you in my professional capacity." To do this faithfully, your undivided attention will be essential in accomplishing the objects you have professed. Your minds must not be distracted by other cares. Your sole business must be the study and practice of dentistry, in its various departments. If other duties are constantly engaging your attention, you must in this proportion have your energies withdrawn from your legitimate business, which of itself is quite sufficient to absorb all the time and talents of any ordinary mind; so that whatever is diverted from it is so much taken from public expectation and rights. The contract will be violated on your part, good faith will be broken; and then complain not if public confidence be withdrawn from you.

And in return, what does the public owe you, and what have you a right to claim from those to whom you devote your time and abilities? They should give you patronage and support in proportion to the faithful manner in which you have rendered your services; and this will, as a general rule, be awarded to you; you can rely upon it with great certainty. A decided preference will generally be shown you over those imperfectly qualified, or who are dividing their time with other pursuits. This patronage may sometimes be tardy; your merits may receive a slow reception; but it must come, it will come, and "according to your faith be it unto you." Like the husbandman who casts his seed in the ground, nothing doubting but that the genial warmth of spring will quicken it, cause it to shoot up, grow, and bring forth an abundant harvest. Yet it may be well to caution you against permitting the spirit of gain to lay hold on your minds. If it does, it will absorb all the finer feelings which cause you to love the profession for its own sake, and a sordid feeling of money-getting will overwhelm you.

A few years of proper application will find you occupying an elevation in public estimation far above those who seemed to outstrip you in the early portion of your career, by a resort to trickery and vain-glorious boasting. There is everything, then, to encourage you in well-doing; and even admitting, (which is hardly possible,) that your success is not commensurate with your merits, still you have the approval of your own consciences, which is worth more to you than gold. Better, far better, remain poor and neglected with a spotless professional character, than to roll in wealth and splendor acquired by ignoble means.

Another matter which relates to the profession, general and special, to the public, and to yourselves, may require your consideration. I mean self-improvement. In the investigation of nature, and in the practice of art, we are constantly progressing; everything relating to our knowledge bears imperfection and a capability of improvement upon its face, and in relation to medicine this is emphatically true. You have, I admit, made attainment sufficient to entitle you to admission into a department of the medical vineyard; but this admission does not imply perfection in this department. You are only advanced students, and are under increased obligation to cultivate the ground you occupy with diligence. The relation you hold in this profession requires that you keep yourselves familiar with physiological and pathological principles. It has been asked, "What has dentistry to do with principles of medicine?—it is only a mechanical trade." Such persons certainly have a very imperfect notion of your position. Is not a knowledge of anatomy necessary in understanding the structure and anatomical connections of the teeth with other portions of the system, particularly the manner of their connection with the nervous and arterial systems? And in order to understand properly these relations, a complete knowledge of the whole circulation, and of the nervous centres with their prolongations, must be familiar. It is impossible to understand the physiological relations of the teeth without a thorough knowledge of anatomy; they are a part of the system affecting and being affected by all of the organs and by every function of the body. Who will not admit the importance of a correct knowledge by every dentist of the physiological influences of the teeth? How else can he explain the sympathetic relations of these organs with other parts of the body? Anatomy and physiology teach us that they receive nerves which are intimately connected with other nerves distributed to important vital organs, and that a mutual action and reaction exists between them. Organs essential to life are also connected with the teeth, in the relation of performing a dependent function, which they cannot properly perform unless the teeth have properly exercised their functions first. The stomach, for instance, relies upon the organs of mastication for a preparation of the food, and unless this is properly accomplished, disease must soon occur in the functions of digestion, from a failure of proper mastication of food. The whole of the organs and functions of the body are so intimately united in one or other of the two modes to which I have alluded, that disease, commencing in any portion, is communicated to other portions; and frequently the beginning of derangement is so slight that it is almost or quite impossible to detect the organ in which the initial step of the morbid process commenced. A knowledge on all points of physiology is therefore absolutely necessary for understanding the pathological relations and dependencies; and what dentist can properly practice his profession without an intimate knowledge of these? It will be impossible for him properly to appreciate the phenomena of disease which is presented to him without it. He would be about as likely to extract a tooth for a sympathetic neuralgia as for caries; toothache with him would be synonymous with caries and extraction. The language of pathology to such an one would be without meaning; the terms inflammation, caries, ulceration, exostosis, &c., would be idle words, expressing nothing of which there was any clear conception in the mind. The elements and processes of inflammation and morbid products, would be to him perfectly unintelligible.

Therapeutic processes could not be applied understandingly or efficiently—in short, the whole practice of a man without the chart and compass of anatomy, physiology, and pathology, would be a mass of confusion and error. We consider it therefore a fact which cannot be subverted, that a knowledge of these sciences, along with general and special therapeutics, is as essential to the dentist as to the general practitioner, and to keep pace with the existing state of knowledge on these subjects is absolutely a necessary duty of the dentist. This range of subjects forms, however, but a small part of the studies which should be cultivated by you. Chemistry, pharmacy, and mechanics, as well as a knowledge of many special diseases and their treatment, must by no means be neglected. Your libraries should be well stored with books on all these subjects; and in addition to these, you ought to take at least one journal and one abstract, devoted to the general profession, and as many devoted to the speciality of dentistry as there are at present in this country.

I have called your attention to the above-named studies, because they are those which you may be the most likely to neglect, on account of their not coming within the immediate range of your every-day duties. Those constant duties which pertain directly to your speciality will not be neglected, of course, by any man having the least claim to professional reputation. In your practice, many diseases of the mouth and adjacent parts must come under your observation, so that your opportunities for studying them may even exceed those engaged in the general practice of surgery. It is your duty to avail yourselves of all these opportunities, and add to the common stock of knowledge whenever it is in your power.

I have now thrown together, very briefly, some few ideas which should engage your attention; it has been done in a hurried and confused manner, at intervals snatched from professional engagements. And, as we are now about to separate, permit me again to impress upon your minds the fact, that upon you must soon devolve the duty and burden of that portion of the profession in which you have cast your lot. The honor and usefulness of the profession of medicine are in part intrusted to you: guard them faithfully, and transmit them to your successors in a bright and improved condition; so that others, seeing your good works, may be led to emulate your example, and you will undoubtedly reap the reward of meritorious professional conduct.

